# Large T_1_ contrast enhancement using superparamagnetic nanoparticles in ultra-low field MRI

**DOI:** 10.1038/s41598-018-30264-5

**Published:** 2018-08-08

**Authors:** Xiaolu Yin, Stephen E. Russek, Gary Zabow, Fan Sun, Jeotikanta Mohapatra, Kathryn E. Keenan, Michael A. Boss, Hao Zeng, J. Ping Liu, Alexandrea Viert, Sy-Hwang Liou, John Moreland

**Affiliations:** 1 National Institute of Standards and Technology, 325 Broadway, CO Boulder, 80305 USA; 2 Nebraska Center for Materials and Nanoscience, University of Nebraska-Lincoln, 855 N.16th St, NE 68588 Lincoln, USA; 3 Department of Physics, University at Buffalo, the State University of New York, 225 Fronczak Hall, NY Buffalo, USA; 4 Department of Physics, University of Texas- Arlington, 502 Yates St, TX 76019 Arlington, USA; 5 Wake Forest Institute for Regenerative Medicine, Wake Forest School of Medicine, NC 27157 Winston-Salem, USA

## Abstract

Superparamagnetic iron oxide nanoparticles (SPIONs) are widely investigated and utilized as magnetic resonance imaging (MRI) contrast and therapy agents due to their large magnetic moments. Local field inhomogeneities caused by these high magnetic moments are used to generate T_2_ contrast in clinical high-field MRI, resulting in signal loss (darker contrast). Here we present strong T_1_ contrast enhancement (brighter contrast) from SPIONs (diameters from 11 nm to 22 nm) as observed in the ultra-low field (ULF) MRI at 0.13 mT. We have achieved a high longitudinal relaxivity for 18 nm SPION solutions, r_1_ = 615 s^−1^ mM^−1^, which is two orders of magnitude larger than typical commercial Gd-based T_1_ contrast agents operating at high fields (1.5 T and 3 T). The significantly enhanced r_1_ value at ultra-low fields is attributed to the coupling of proton spins with SPION magnetic fluctuations (Brownian and Néel) associated with a low frequency peak in the imaginary part of AC susceptibility (*χ*”). SPION-based T_1_-weighted ULF MRI has the advantages of enhanced signal, shorter imaging times, and iron-oxide-based nontoxic biocompatible agents. This approach shows promise to become a functional imaging technique, similar to PET, where low spatial resolution is compensated for by important functional information.

## Introduction

Magnetic resonance imaging (MRI) systems are widely used for clinical diagnostics and are typically performed in a high magnetic field from 1.5 T to 7 T. High fields are preferred to achieve sufficient signal required for short imaging times (less than a few minutes) and high spatial resolution (less than a millimeter). Contrast agents are widely used in clinical MRI, to enhance image contrast by either increasing (positive contrast)^1^ or decreasing (negative contrast)^2–4^ the local magnetic resonance (MR) signal. This is typically done by locally reducing T_1_ or T_2_, for positive and negative contrast respectively, where T_1_ is the longitudinal and T_2_ is the transverse proton spin relaxation time. T_1_ relaxation comes from the fluctuating electron spin moment of the contrast agent coupling with neighboring water protons inducing energy transfer^5^. The proton spins that are very near the contrast agent quickly relax back to their equilibrium magnetization, leading to a shortened T_1_ and an enhanced MR signal since the proton magnetization fully recovers between excitation pulses. In contrast, T_2_ relaxation comes from long-range magnetic dipolar fields of the magnetic particles that shift the proton resonance frequency locally and dephase the proton spin precession, leading to a short T_2_ and a reduced MR signal^5^. In this letter we show that magnetic nanoparticles, which give large negative contrast in high field MRI, give very large positive contrast in ultra-low field (ULF) MRI and demonstrate that, by tailoring the magnetic nanoparticle fluctuation rate, agents with much higher visibility can be obtained.

Superparamagnetic iron oxide nanoparticles (SPIONs) have enhanced magnetic moment, typically by a factor of ~1000, compared to conventional Gd-based paramagnetic agents, leading to large relaxation effects^6–8^. At clinical MRI fields (1.5 T or 3 T), T_1_ reduction for SPIONs is small since magnetic fluctuations are suppressed while T_2_ reduction is large due to the nearly saturated time-average moment of SPIONs and the large local volume over which water protons are dephased^2–4^. SPIONs with negative contrast have been used for detection of hepatocellular carcinoma^9,10^, prostate cancer^11^, sentinel lymph node localization in breast cancer^12^, diagnosis of cardiovascular disease^13^, identifying inflamed carotid plaques^14,15^ and cell tracking^16^. Even though T_2_ suppression of SPIONs can improve the imaging contrast by reducing the MR signal, T_1_ agents are preferred since they enhance signal, preserve the underlying tissue signal, and shorten the imaging times. T_1_ contrast enhancement has been reported at high magnetic fields for certain SPIONs^17–20^ including ultra-small-size Fe_3_O_4_ nanoparticles (<3 nm)^18^, Gd doped Fe_3_O_4_ nanoparticles (4.8 nm)^19^ and Fe_3_O_4_ nanoplates^20^. In some cases the relaxation is believed to be due to surface-spin canting effects.

Here, we present an alternative approach for achieving T_1_ contrast enhancement from SPIONs by going to ultra-low magnetic fields. In ULF MRI systems, the operating magnetic field *B*_0_ is around 0.13 mT, corresponding to a resonance frequency of *f*_0_ = 5.56 kHz, which is four orders of magnitude lower than a typical clinical MRI field of 1.5 T (*f*_0_ = 63.87 MHz)^21–25^. At ultra-low field, the magnetic moment of the SPIONs is not saturated, and they have large spin fluctuations. T_1_ (positive) contrast can be greatly enhanced since the proton Larmor precession period is comparable with the relaxation times of magnetic nanoparticles^26–29^, leading to a large increase in the proton spin longitudinal relaxivity r_1_. The proton spin relaxivities, r_1_ and r_2,_ are the change in relaxation rates R_1_ and R_2_ per unit Fe concentration, respectively, where R_1_ = 1/T_1_, R_2_ = 1/T_2_. In addition, the smaller non-saturated magnetic moment of the nanoparticle reduces the transverse relaxivity, r_2_, compared to the high field r_2_, minimizing undesirable negative contrast.

## Results

### Magnetic resonance measurements

**Figure 1:**
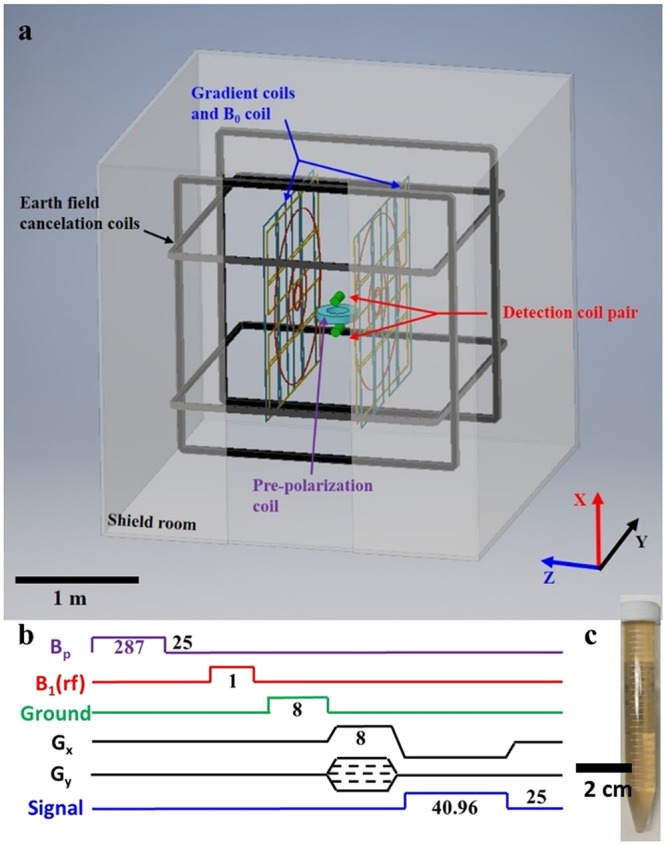
(**a**) ULF MRI system showing differential detector coil, *B*_1_(rf), (red) on either side of the shielded pre-polarization field, *B*_p_, (purple) with applied magnetic field, *B*_0_, (blue). (**b**) ULF MRI gradient echo (GRE) sequence. Each pulse duration is marked in milliseconds (TR = 400 ms; TE = 27 ms). (**c**) Photograph of 16 mL vials used in ULF MRI imaging phantom.

**Figure 2:**
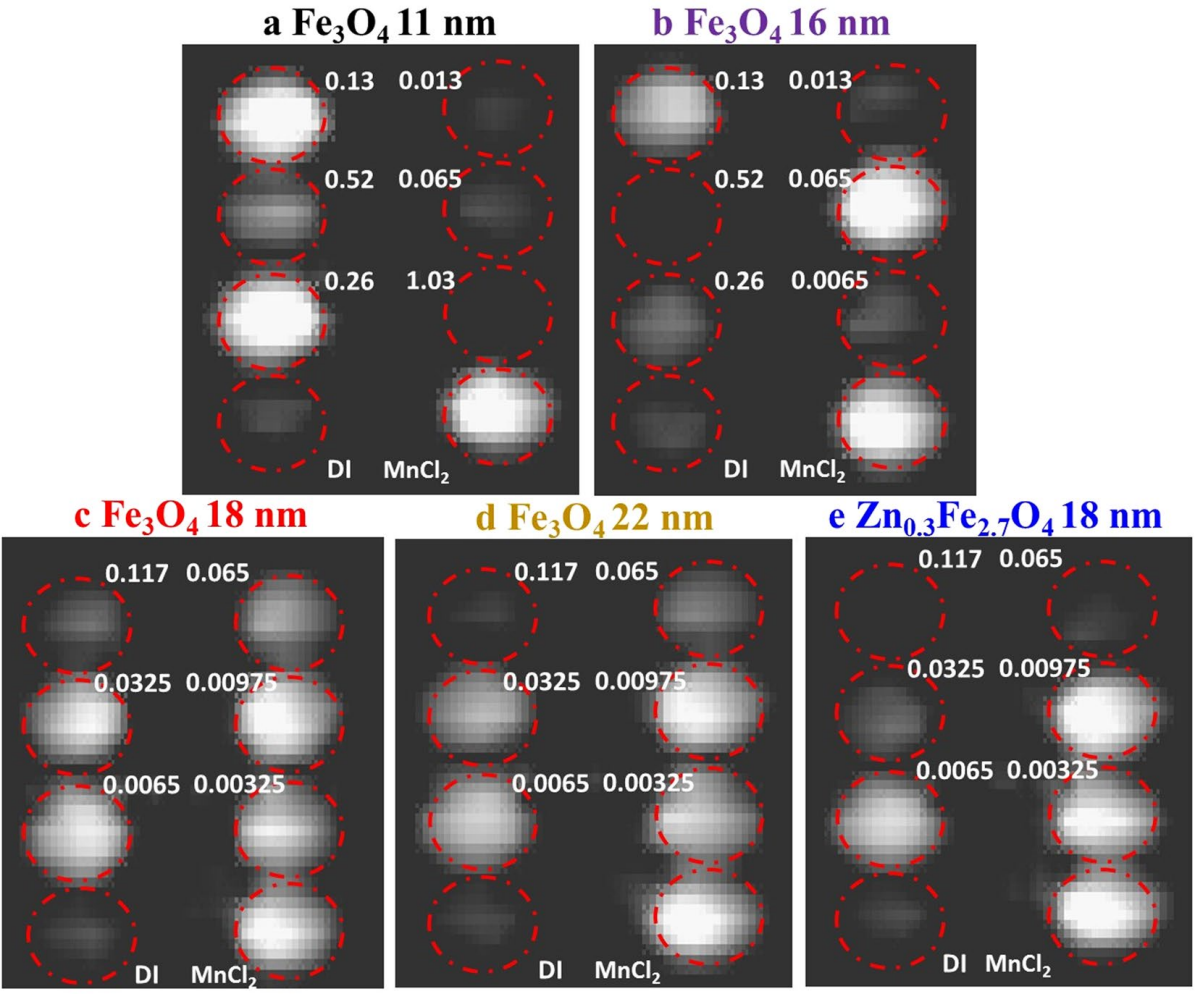
ULF MRI 2D images of different SPION concentrations in 16 mL vials with a vial diameter of 14 mm: (**a**) 11 nm Fe_3_O_4_ solutions, (**b**) 16 nm Fe_3_O_4_ solutions, (**c**) 18 nm Fe_3_O_4_@SiO_2_ solutions, (**d**) 22 nm Fe_3_O_4_@SiO_2_ solutions, and (**e**) 18 nm Zn_0.3_Fe_2.7_O_4_@SiO_2_ solutions. The Fe concentrations of the solutions (in mM) are listed in the figures. DI water and MnCl_2_ (0.167 mM Mn in DI water) control samples are placed at the bottom of each image for normalization and comparison.

**Figure 3:**
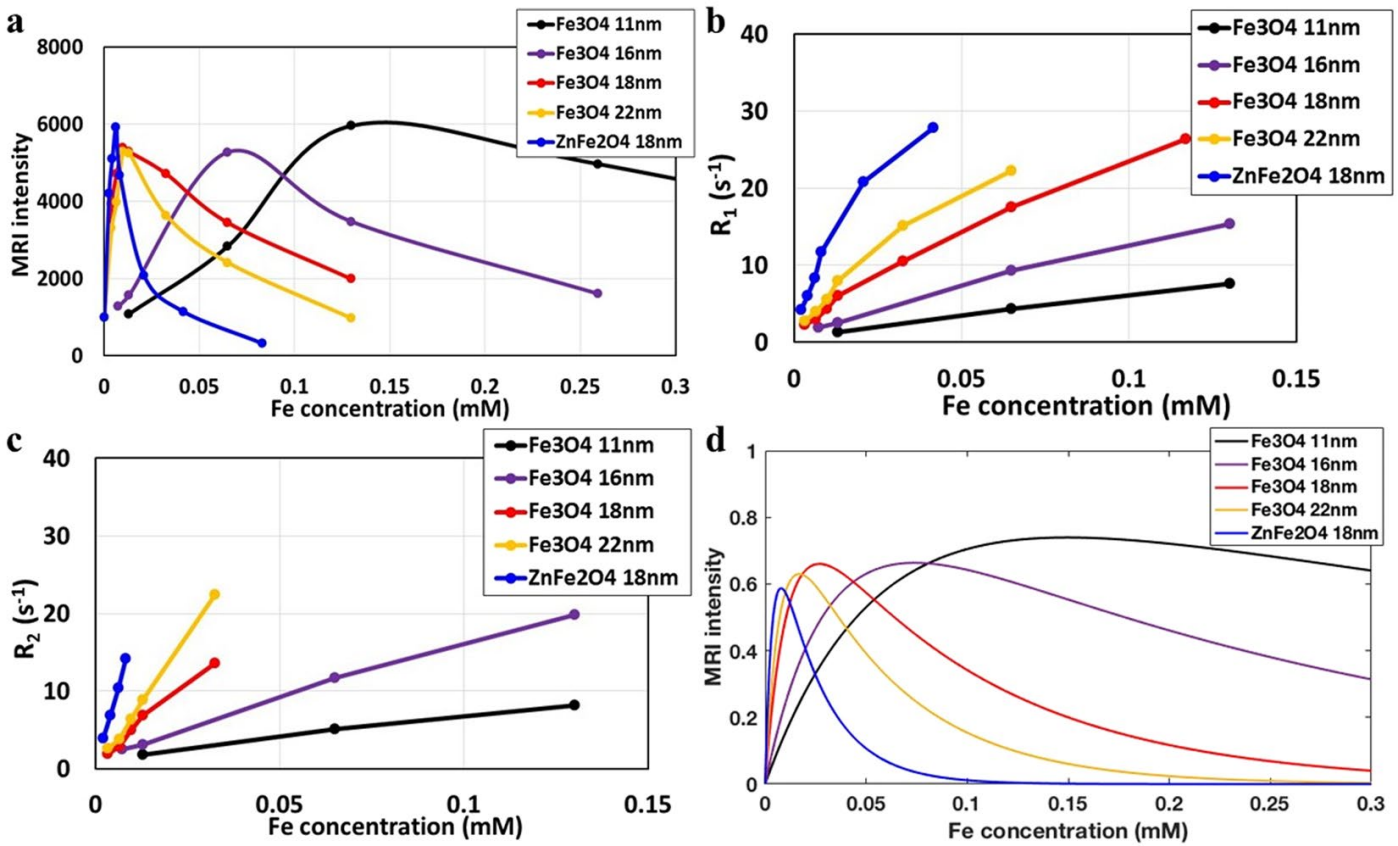
(**a**) The 0.13 mT MRI signals for different particle sizes versus Fe concentration are plotted in black dots (Fe_3_O_4_ 11 nm), purple dots (Fe_3_O_4_ 16 nm), red dots (Fe_3_O_4_@SiO_2_ 18 nm), yellow dots (Fe_3_O_4_@SiO_2_ 22 nm), and blue dots (Zn_0.3_Fe_2.7_O_4_@SiO_2_ 18 nm) (**b**) Longitudinal relaxation rate (R_1_) versus Fe concentration of the solution in solid dots. (**c**) Transverse relaxation rate (R_2_) versus Fe concentration for the solution in solid dots. (**d**) Simulation of MRI signal versus Fe concentration with corresponding r_1_ and r_2_ relaxivity of each nanoparticles and TR = 400 ms, TE = 27 ms. (Lines included to help guide the eye.)

MR measurements were performed in a low-cost ULF MRI system with a Faraday coil detector and a bilateral planar coil array that includes the *B*_0_ coil and the gradient field (*G*_*x*_, *G*_*y*_ and *G*_*z*_) coils. The earth’s field was cancelled by two pairs of Helmholtz coils (*B*_*x*_ and *B*_*y*_), as shown in Fig. 1(a). To generate a measurable signal for the ULF MRI scanner, a pre-polarization field pulse (34.5 mT) is applied to enhance the proton magnetization before the MR pulse sequence. For imaging, we used a typical T_1_-weighted ULF MRI gradient echo (GRE) pulse sequence with repetition time (TR) of 400 ms, and gradient echo time (TE) of 27 ms as shown in Fig. 1(b). Five types of SPION solutions were used: 11 nm Fe_3_O_4_ and 16 nm Fe_3_O_4_ nanoparticles with citric acid surface modification, and 18 nm Fe_3_O_4_@SiO_2_, 22 nm Fe_3_O_4_@SiO_2_ and 18 nm Zn_0.3_Fe_2.7_O_4_@SiO_2_ nanoparticles with a thin shell surface coating of SiO_2_. The dimensions listed are the average core diameter. The synthesis processes along with transmission electron microscopy images are described in the Methods section and in references^30–32^. SPIONs were diluted with deionized (DI) water and transferred to 16 mL sample vials for ULF MRI imaging. The particle concentrations range from 0.25 µg/mL to 40 µg/mL by weight corresponding to Fe concentrations ranging from 0.00325 mM to 0.52 mM. A photograph of a sample vial with SPION solution is shown in Fig. 1(c). T_1_-weighted ULF MRI 2D GRE images are obtained with a pixel size of 2.8 mm × 3.6 mm with imaging duration of 1.6 hours, which is slower than that of superconducting quantum interference device (SQUID) detected ULF MRI (due to our lower signal to noise ratio) but sufficiently fast enough for the study here. The ULF MRI showed significant differences among five types of SPION solutions in Fig. 2(a–e). The ULF MRI signal versus Fe concentration for each SPION solutions are plotted in Fig. 3(a) (with the signal normalized to the DI water signal set to an arbitrary intensity of 1000). The ULF MRI signal initially increases (positive contrast) with increasing Fe concentration, at very low Fe concentration, due to the suppression of T_1_, while at high Fe concentration the signal decreases (negative contrast) due to shorter T_2_ times. This leads to a peak in the signal *vs*. Fe concentration; similar phenomenon has been observed in regular Gd-based T_1_ contrast agents^33^. To achieve the same T_1_-weighted ULF MRI signal, the required Fe concentration for the 18 nm Zn_0.3_Fe_2.7_O_4_@SiO_2_ nanoparticles is one tenth of that required for the 11 nm Fe_3_O_4_ nanoparticles.

### Pulse sequence and relaxivities

T_1_ of the SPION solutions is measured with a prepolarization evolution pulse sequence^34,35^. In our setup *B*_0_ = 0.13 mT and T_1_ = 2.7 s for DI water and T_1_ = 0.12 s for the MnCl_2_ solution with Mn concentration of 0.167 mM. These results are comparable with SQUID based ULF MRI system of *B*_0_ = 0.128 mT, as reported in ref.^35^. R_1_ and R_2_ are linearly increasing as a function of the Fe concentration of five SPION solutions as shown in Fig. 3(b,c). The r_1_, r_2_, r_2_/r_1_ ratio, and ULF MRI maximum signal for the five SPION solutions are listed in Table 1. The important figures of merit for positive contrast are a large r_1_ value and a low r_2_/r_1_ ratio. This insures a large signal increase at low Fe concentrations. The 18 nm Zn_0.3_Fe_2.7_O_4_@SiO_2_ nanoparticles show a very high r_1_ = 615 s^−1^ mM^−1^, with only a modest increase in the r_2_/r_1_ = 2.7. In contrast, commercial high-field Gd-based T_1_ agents have r_1_ = 3 to 7 s^−1^ mM^−1^, with r_2_/r_1_ ~ 1.2 to 1.5^1^.

**Table 1:** SPION relaxivities in s^−1^ mM^−1^ at 0.13 mT, where mM refers to the Fe concentration, and normalized MRI intensities.

	r_1_	r_2_	r_2_/r_1_	ULF MRI intensity
11 nm Fe_3_O_4_	45	54	1.2	6.1
16 nm Fe_3_O_4_	78	143	1.8	5.2
18 nm Fe_3_O_4_@SiO_2_	212	397	1.9	5.3
22 nm Fe_3_O_4_@SiO_2_	316	692	2.2	5.3
18 nm Zn_0.3_Fe_2.7_O_4_@SiO_2_	615	1657	2.7	5.9

The MR signal is proportional to the transverse proton magnetization, *M*_*t*_, at an echo time TE when the signal is acquired. *M*_*t*_ is a function of the pulse sequence parameters TR, TE and Fe concentration-dependent relaxation rates R_1_, R_2_ as described by the standard signal equation^36^

1 $$\begin{array}{c}{M}_{t}({C}_{Fe})={e}^{-TE\cdot {R}_{2}({C}_{Fe})}\cdot sin\alpha \cdot \frac{1+{e}^{-TR\cdot {R}_{1}({C}_{Fe})}-2{e}^{-(TR-\frac{TE}{2})\cdot {R}_{1}({C}_{Fe})}}{1+{e}^{-TR\cdot {R}_{1}({C}_{Fe})}\cdot cos\alpha }\end{array}$$

where C_Fe_ is Fe concentration, and α = π/2 is flip angle of rotation. We use Eq. 1 to simulate how the detected proton spin magnetization varies as a function of Fe concentration in the different SPION solutions, plotted in Fig. 3(d), and obtained good fits to our experimental data using the implemented pulse sequence parameters and measured relaxation rates. The highest value of r_1_ (615 s^−1^ mM^−1^) gives a 6 times enhanced MRI signal at low Fe concentrations (0.01 mM), which implies that ULF MRI may provide a technology for functional imaging using specially designed high-visibility SPIONs giving positive contrast.

### The r1 and r2 values at high fields (3 T)

We measured the r_1_ and r_2_ values at high fields (3 T) using a standard inversion recovery sequence with variable inversion times for T_1_ and a spin echo sequence with variable echo time for T_2_. For the 16 nm Fe_3_O_4_ solutions we found that r_1_ = 11 s^−1^ mM^−1^ and r_2_ = 137 s^−1^ mM^−1^ (r_2_/r_1_ ratio of 12.5). After the high field MRI scan, the SPION solutions are sonicated and measured again with ULF MRI, and a reduction of the ULF MRI signal was observed, indicating that agglomeration of the SPIONs took place during the high-field MRI scan. Cluster or chaining of magnetic nanoparticles in large magnetic fields, due to their strong magnetic dipolar attraction, is a major limitation of high-field agents putting limits on the size of magnetic moments that can be utilized and creating stringent requirements on surface ligands to provide adequate separation. In comparison, repeat images in the ULF scanner showed no agglomeration effects, which is a significant advantage of ULF-MRI based nanoparticle contrast imaging.

### Magnetic properties of nanoparticles and AC susceptibility

**Figure 4:**
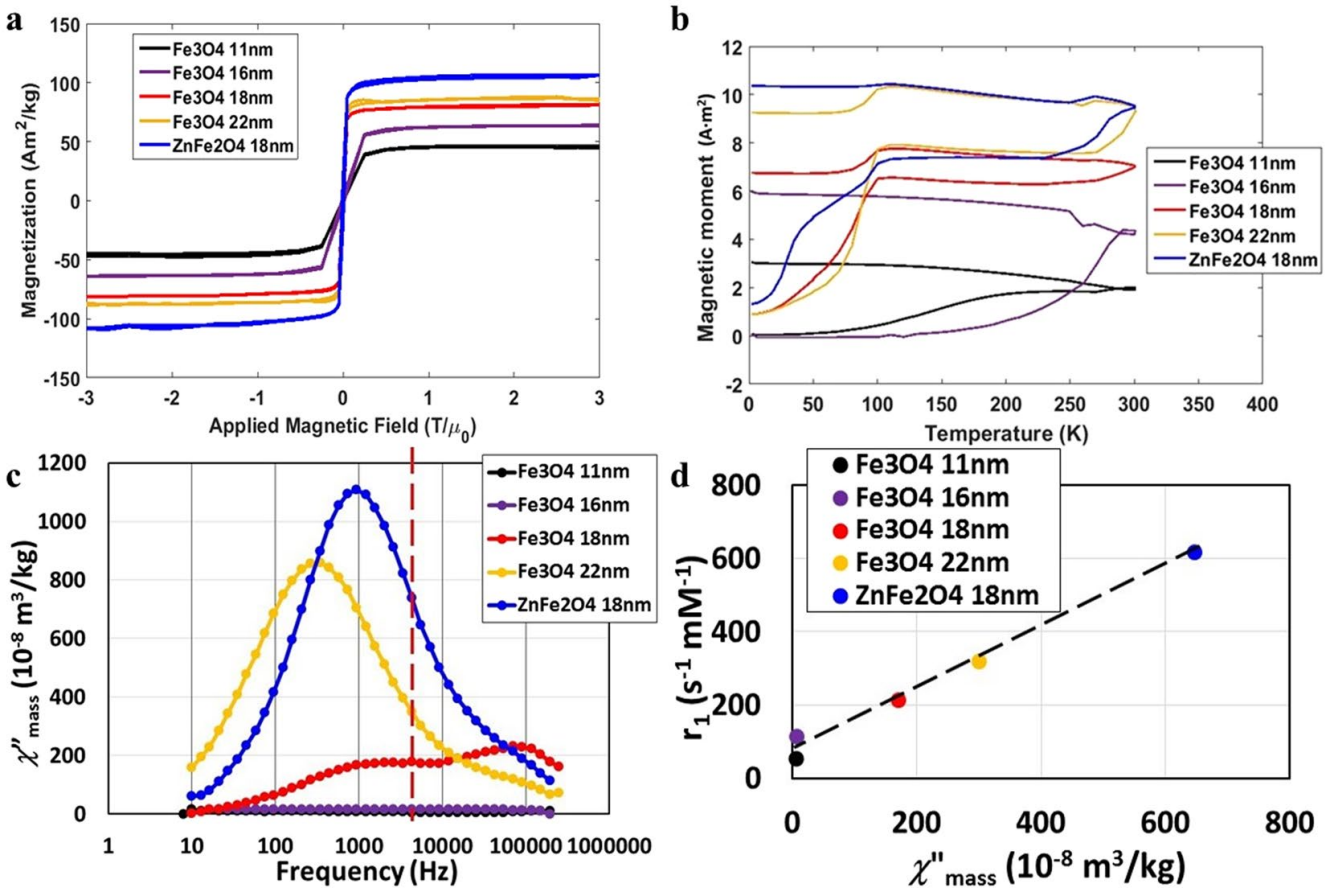
Magnetic properties of magnetic nanoparticle solutions with concentrations of 1000 μg/mL, with Fe_3_O_4_ 11 nm (black dots), Fe_3_O_4_ 16 nm (purple dots), Fe_3_O_4_@SiO_2_ 18 nm (red dots), Fe_3_O_4_@SiO_2_ 22 nm (yellow dots) and Zn_0.3_Fe_2.7_O_4_@SiO_2_ 18 nm (blue dots). (**a**) Magnetization versus applied magnetic field at room temperature. (**b**) Zero field cooled-field cooled curves with field strength of 20 mT, (**c**) Imaginary part of AC mass susceptibility (χ”) versus frequency response at 293 K. The dashed line indicates ULF MRI operating frequency of 5.56 kHz. (**d**) r_1_ vs. χ” at 5.56 kHz for the five nanoparticles sizes. (Lines included to help guide the eye).

The room temperature magnetization versus applied magnetic field and zero field cooled/field cooled (ZFC/FC) loops of five SPION solutions are shown in Fig. 4(a,b). The closed Langevin-like hysteresis loops and low blocking temperatures (the temperature above which thermal energy overcomes anisotropy energy, causing the net moment of the particle to fluctuate randomly) indicate that all five types of nanoparticles are superparamagnetic at room temperature. The blocking temperatures of Fe_3_O_4_ nanoparticles are not linear with particle volume, which is possibly due to different magnetic anisotropy energy densities resulting from different synthetic conditions.

To better understand the T_1_ contrast enhancement mechanisms in SPION solutions at ultra-low field, we measured the imaginary part of the magnetic AC mass susceptibility (*χ*”) from 10 Hz to 250 kHz for five SPION solutions, shown in Fig. 4(c). The peak in *χ*” is attributed to either Brownian rotation of the nanoparticle or Néel relaxation of nanoparticle moment and occurs at a frequency that is given by the inverse of the nanoparticle moment relaxation time^37–39^. The magnetic fluctuation spectra are proportional to *χ*” through the fluctuation-dissipation theorem. We therefore expect strong proton spin T_1_ relaxation when proton resonant frequency (shown as the dashed vertical line in Fig. 4(c)) is near peaks in *χ*”, where nanoparticle magnetic fluctuation spectral density is large. We found that the *χ*” spectrum is flat for the 11 nm Fe_3_O_4_ solution compared to a higher frequency *χ*” peak observed at 198 kHz in the 16 nm Fe_3_O_4_ solution and at 100 kHz in the 18 nm Fe_3_O_4_@SiO_2_ solution. In comparison, a lower frequency *χ*” peak is observed at 1000 Hz in the 18 nm Fe_3_O_4_@SiO_2_ solution, at 250 Hz in the 22 nm Fe_3_O_4_@SiO_2_ solution and at 1000 Hz in the 18 nm Zn_0.3_Fe_2.7_O_4_@SiO_2_ solution. We calculated the Néel relaxation frequency of 18 nm Fe_3_O_4_@SiO_2_ with the measured magnetic properties and confirmed that the higher frequency of relaxation is Néel relaxation, and the lower frequency relaxation is Brownian relaxation as described in the Materials and Methods section.

We plot the measured r_1_ versus *χ*” at the proton resonance frequency of *f*_0_ = 5.56 kHz for the five different nanoparticles in Fig. 4(d). Also shown is a linear fit of r_1_ versus *χ*”(*f*_0_) showing a strong linear correlation. The large and tunable magnetic susceptibility of SPIONS cannot be obtained at high magnetic fields and is a unique attribute of ULF MRI.

## Discussion

In SPION solutions, the longitudinal relaxation behavior is due to the relative motion of the water protons near the particle and the magnetic fluctuations of the nanoparticle through physical movement (Brownian translation and rotation) as well as intrinsic fluctuations of magnetic moment (Néel relaxation). According to the fluctuation dissipation theorem, the nanoparticle magnetic fluctuation spectral density should be proportional to the imaginary part of the magnetic susceptibility. These magnetic moment fluctuations give rise to fluctuating magnetic fields experienced by the water protons and lead to strong T_1_ relaxation of the proton spins. As seen in Fig. 4(c), it is possible to engineer the magnetic susceptibility and hence the magnetic fluctuations of the nanoparticles, over a wide range of frequencies. While the best sample studied here, the 18 nm Zn_0.3_Fe_2.7_O_4_@SiO_2_, had a peak close to the proton resonance frequency, if we adjust the particle properties or the resonant frequency we could get significant additional enhancement in T_1_ relaxivity and agent induced positive contrast. To obtain a high r_1_ value, we can tune either the magnetic properties of nanoparticle (moment and magnetic anisotropy energy) or mechanical properties (hydrodynamic shape and volume) to couple the *χ*” peak with the operating frequency of the ULF MRI. Although our highest r_1_ value is due to Brownian relaxation, which may be hindered in more biologically realistic viscous media, Néel relaxation (observed in 16 nm Fe_3_O_4_ nanoparticles) still gives appreciable contrast enhancement and can be tuned by engineering the magnetic anisotropy energy. Engineering Néel fluctuations can be done by changing either material properties, size, or shape of the particles, and is advantageous because this contrast will be independent of local tissue environment. Engineering the Brownian fluctuations has the advantage that the agents will give contrast only in special tissue environments (e.g. low viscosity environments) and the contrast may be turned on or off when binding or unbinding from a target.

ULF-MRI with high-visibility positive contrast agents provides a combination of good soft tissue contrast and functional imaging that is more deployable in a surgical suite or a doctor’s office. With new high-sensitivity optical magnetometers^40^ and advanced machine-learning based image reconstruction techniques^41^ coming online, radically new designs are possible with local field probes and coils. Viewing magnetic nanoagents as low frequency stochastic oscillators that strongly couple to nuclear spins, provides a path to greatly increase nanoparticle visibility. These systems may have unique applications for cell tracking, drug delivery monitoring, sentinel lymph node monitoring, and interventional procedures.

In summary, a 6-times T_1_ contrast enhancement for the 18 nm Zn_0.3_Fe_2.7_O_4_@SiO_2_ solution was achieved using room-temperature ULF MRI system. An extremely high value of r_1_ of 615 s^−1^ mM^−1^, over 100 times larger than commercial high field T_1_ agents, has been achieved. A linear relationship of r_1_ versus *χ*” at 5.56 kHz, the proton resonance frequency, was observed among all five different SPION solutions, which can be attributed to the magnetic fluctuations of the nanoparticles that relate to either Brownian motion or Néel relaxation. The ability to have large tunable magnetic susceptibility in SPIONS is a unique feature of ULF MRI and cannot be obtained in conventional high field MRI. The ULF MRI platform, combined with the use of designed SPIONs as high visibility positive contrast agents, therefore, creates new possibilities for safe inexpensive functional imaging.

## Methods

### Magnetic nanoparticle synthesis

**Figure 5:**
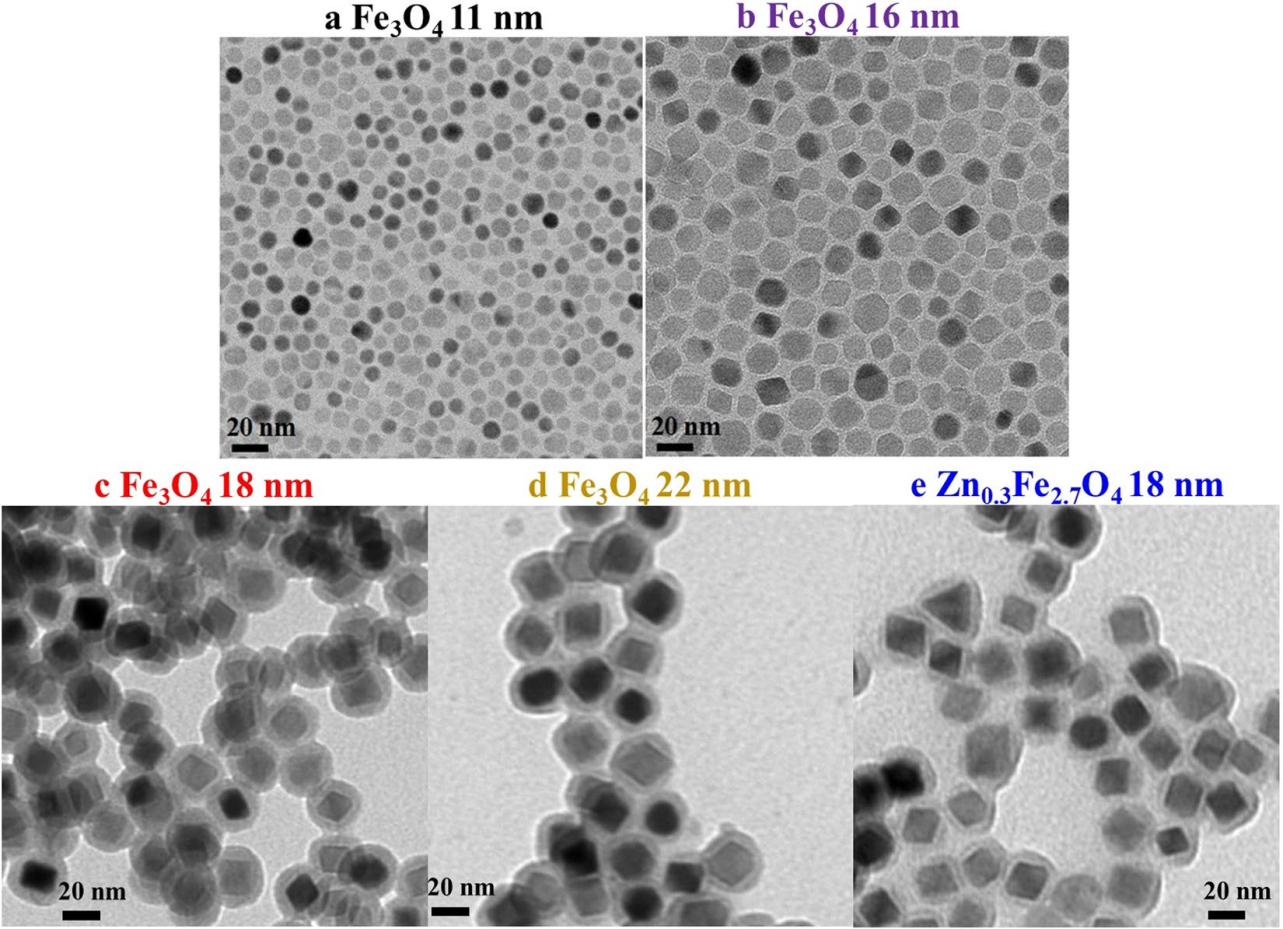
TEM images of magnetic nanoparticles with 20 nm scale bar. (**a**) Fe_3_O_4_ nanoparticles with diameter of 11 nm, (**b**) Fe_3_O_4_ nanoparticles with diameter of 16 nm, (**c**) Fe_3_O_4_@SiO_2_ nanoparticles with 18 nm core diameter and 5 nm silica shell thickness, (**d**) Fe_3_O_4_@SiO_2_ nanoparticles with 22 nm core diameter and 5 nm silica shell thickness, (**e**) Zn_0.3_Fe_2.7_O_4_@SiO_2_ nanoparticles with 18 nm core diameter and 5 nm silica shell thickness. The ordering and agglomeration of the nanoparticles are due to the interactions when the suspended particles are dried on the TEM grids. The nanoparticles are dispersed during ULF-MRI relaxivity measurements.

The SPIONs have a magnetite structure (Fe_3_O_4_) as confirmed by X-ray diffraction (XRD) and transmission electron microscope (TEM)^30^. TEM images of the nanoparticles used in this study are shown in Fig. 5. In a typical synthesis of 11 nm Fe_3_O_4_ nanoparticles, a mixture of 5.0 mmol of iron acetylacetonate and 15.0 mmol of oleylamine, 3.0 mmol of oleic acid was heated at 200 °C under Ar atmosphere for 4 h. The black colored precipitate was subjected to magnetic separation and washed with a mixture of hexane and acetone several times to remove any uncoordinated amine and acid molecules. The 16 nm Fe_3_O_4_ nanoparticles were obtained with the increase of oleic acid concentration to 7.5 mmol. Finally, these amine-capped magnetic nanoparticles were dried at normal ambient conditions. To use these samples as MRI contrast agent, as-prepared Fe_3_O_4_ nanoparticles were subjected to surface modification with citric acid. 200 mg of the as-prepared Fe_3_O_4_ nanoparticles were dispersed in 20 mL of toluene and mixed with 20 mL of dimethylformamide (DMF) containing 20 mM of citric acid. Under Ar ambient, the mixture was then continuously stirred at 80 °C for 8 h. The final product was subjected to magnetic separation and was washed with ethanol several times to remove uncoordinated citric acid molecules. The SPIONs are diluted with deionized (DI) water and the particle concentrations ranges from 0.5 µg/mL to 200 µg/mL (Fe concentration from 0.0065 mM to 2.6 mM).

Nanoparticles with silica shells and core size of 18 nm Fe_3_O_4_@SiO_2_, 22 nm Fe_3_O_4_@SiO_2_, and 18 Zn_0.3_Fe_2.7_O_4_@SiO_2_ were synthesized by a one-pot solution method through thermal decomposition of a mixture of metal acetylacetonates with surfactants in a high-boiling point organic solvent^31^. Under a gentle flow of Ar, Iron(III) acetylacetonate (2.7 mmol), zinc(II) acetylacetonate (0.3 mmol), sodium oleate (2 mmol) and oleic acid (4 mL) were mixed with benzyl ether (20 mL). The mixture was magnetically stirred under a flow of Ar and then heated to 120 °C for 1 h. Under an Ar blanket, the solution was further heated to reflux (~300 °C) and kept at this temperature for 1 h. The mixture was then cooled down to room temperature by removing the heating mantle. The sizes of nanoparticles were tuned by controlling the heating rate during heating from 120 °C to 300 °C. The silica shells were coated on the hydrophobic nanoparticles via a reverse microemulsion method. The silica coating makes the nanoparticles hydrophilic, leading to aqueous dispersions stable for several years without agglomeration.

### Néel/ Brownian relaxation modeling and connection between T_1_ and susceptibility

For application development, the Brownian and Néel relaxation characteristics of magnetic nanoparticles in solution need to be tailored by adjusting particle diameter, composition, and anisotropy (shape and/or crystalline). The dynamic response of a magnetic nanoparticle in solution is described by the complex magnetic susceptibility $\,\overrightarrow{M}(\omega )=\overline{\overline{\chi }}\overrightarrow{{\rm{H}}}(\omega )$ where $\overrightarrow{M}$ is the moment per unit volume and $\overrightarrow{{\rm{H}}}$ is the magnetic field in A/m. At low frequencies, the susceptibility has a Debye form characterized by exponential relaxation times τ_*B,N*_ and corresponding relaxation frequencies $2\pi {f}_{B,N}=\frac{1}{{\tau }_{B,N}}$, where the imaginary component of the susceptibility peaks. Néel relaxation dominates when the particle is fixed, and the magnetic moment rotates within the particle. Brownian relaxation dominates when the particle is free to rotate, and the magnetic moment is locked to the internal lattice. For *in vivo* biomedical applications, magnetic nanoparticles may be lodged in highly viscous tissues that reduce the Brownian rotation. The particle rotation needs to overcome the surface friction in a viscous fluid, which happens at a rotational frequency^38,39^

2 $$\begin{array}{c}{f}_{B}=\frac{{k}_{B}T}{8\cdot {\pi }^{2}\cdot \eta \cdot {{R}_{H}}^{3}}\end{array}$$

where, *k*_*B*_ is Boltzmann’s constant, *T* is the temperature, *η* is the viscosity of the fluid and *R*_*H*_ is the hydrodynamic radius of the nanoparticle. In the case of Néel relaxation the magnetic moment needs to overcome the anisotropy energy barrier and the fluctuation rate is given by

3 $$\begin{array}{c}{f}_{N}={f}_{0}\cdot exp(-\frac{{K}_{a}V}{{k}_{B}T})\end{array}$$

where *K*_*a*_ is the anisotropy energy density, *V* is the particle volume, and *f*_0_ is the attempt frequency, here chosen to be 1 GHz. The *K*_*a*_ of the particle is typically not that of the bulk material rather an effective *K*_*a*_, influenced by the particle size, surface anisotropy, and magnetite core composition in combination, must be determined. These parameters are controlled using different synthesis processes. The effective anisotropy energy can be estimated from blocking temperature (T_B_) determined from the ZFC-FC measurements,

4 $$\begin{array}{c}{T}_{B}=\frac{{K}_{a}V}{{k}_{B}\cdot \,{ln}(\tau \cdot {f}_{0})}\end{array}$$

where τ is the measurement time.

The extracted *K*_*a*_ for 18 nm Fe_3_O_4_@SiO_2_ nanoparticle solutions, based on blocking temperature data (Fig. 4(b)) is *K*_*a*_ = 0.15 × 10^5^ J/m^3^ and *K*_*a*_*V/k*_*B*_*T* ≈ 3.4 at zero field and room temperature, assuming small variation of *K*_*a*_ from the measurement temperature to room temperature. The calculated Néel relaxation frequency (Eq. 1) is *f*_*N*_ = 134 kHz. The relaxation spectra (Fig. 4(c)) shows two peaks: one at 100 kHz and the other at 1 kHz corresponding to Néel and Brownian relaxation processes respectively. The Néel relaxation frequency of the 18 nm Fe_3_O_4_@SiO_2_ nanoparticle is too high to couple with proton Larmor frequency of current ULF MRI, but it could have potential imaging enhancement at low field MRI operating around 3 mT. Alternatively, one can increase *K*_*a*_ by doping^29^ or increase the particle volume to slow down the fluctuations.

There is an intrinsic connection between magnetic fluctuations and the imaginary part of the magnetic susceptibility given by the fluctuation-dissipation theorem^42^:

5 $$\begin{array}{c}{\mu }_{0}{\int }_{-{\rm{\infty }}}^{{\rm{\infty }}}\langle {M}_{\nu }(t){M}_{\mu }(t=0)\rangle {e}^{i\omega t}dt=\frac{\hslash }{V}coth(\frac{\hslash \omega }{2{k}_{B}T}){\chi }^{{\rm{^{\prime} }}{\rm{^{\prime} }}}\nu \mu (\omega )\end{array}$$

The magnetic fluctuations give rise to local field fluctuations that cause T_1_ relaxation, which in the simplest model^43^ is given by:

6 $$\begin{array}{c}\frac{1}{{T}_{1}}=4{\gamma }_{I}^{2}{b}^{2}\frac{{\tau }_{c}}{1+{\omega }^{2}{\tau }_{c}^{2}}\,\end{array}$$

where $\,{{\boldsymbol{\gamma }}}_{{\boldsymbol{I}}}$ is the proton gyromagnetic ratio, *b* is the fluctuating magnetic field amplitude, ${{\boldsymbol{\tau }}}_{{\boldsymbol{c}}}$ is the relaxation time of the field fluctuations. To obtain large T_1_ relaxation, one needs the relaxation frequency to be close to the proton resonance frequency and have a large fluctuation field amplitude. Magnetic nanoparticles only have large fluctuations at low fields, where the Zeeman coupling between the particles’ moment and applied field is sufficiently small relative to thermal energy. At larger fields, Zeeman coupling dominates and the particles become saturated; that is, the net moment of each particle is locked in a unique orientation.

### ULF MRI pulse sequences

The imaging MR pulse sequence is illustrated in Fig. 2(b); a pre-polarization field of 34.5 mT pre-aligns the water proton spins to provide a manageable MR signal that can be detected by the Faraday-coil. An AC magnetic field (*B*_*1*_), which drives protons at the Larmor frequency is applied for 1 ms to tip the spins away from *B*_0_ by 90 degrees. An 8 ms “grounding pulse” enables a relay that shorts the detector coil to ground to prevent ring-down noise from saturating the preamplifier. The repetition time (TR) is 400 ms, and the echo time (TE) is 27 ms.

For T_1_ measurements, a static field *B*_*0*_ = 0.13 mT is applied continuously. The prepolarizing field *B*_*p*_ = 20 mT is turned on for a period *t*_*p*_ = 287 ms and switched off adiabatically, allowing the spins to relax for an evolution time *t*_*ev*_ in *B*_0_, before a standard spin-echo imaging sequence is applied. *t*_*ev*_ is varied between 0 s and 4 s, depending on the sample. The signal is proportional to the longitudinal magnetization after it has relaxed for a time *t*_*ev*_. The observed signal versus *t*_*ev*_ is fit to a simple exponential decay model to obtain T_1_.

For T_2_ measurements, a static field *B*_0_ = 0.13 mT is applied continuously, and the prepolarizing field *B*_*p*_ = 20 mT is turned on for a period *t*_*p*_ = 287 ms. The evolution time *t*_*ev*_ = 22 ms is now fixed, after which a 90-degree tipping pulse is applied, followed at a time TE/2 by a 180-degree refocusing pulse. The free induction decay is acquired at a time TE and is proportional to the transverse magnetization after it has decayed for a time TE. TE is varied, and the observed signal versus TE is fit to a simple exponential decay model to obtain T_2_.

The relaxivities are then calculated by plotting R_1_ = 1/T_1_, R_2_ = 1/T_2_ vs Fe concentration, using the slope of a linear fit to the data.

### Magnetic properties measurement

Magnetization versus applied magnetic field (M-H) loops and zero field cooled-field cooled (ZFC-FC) loops are measured using a SQUID magnetometer, as shown in Fig. 3(a,b). The SPION solutions were heat-sealed in polypropylene containers with a particle concentration of 1.0 mg/mL and a mass of about 160 mg. The diamagnetic components are subtracted from the M-H loops using the known mass of the water and sample container. The ZFC-FC loops are measured using the same SPION samples under presence of a 20 mT magnetic field after the initial zero field cooling phase. As shown in Fig. 4(b), the room temperature imaginary part of AC mass susceptibilities of the SPION solutions were measured with a commercial AC susceptometer using SPION solutions with a particle concentration of 1.0 mg/mL, a mass of approximately 180 mg, and an applied AC field of 398 A/m. The ZFC-FC and the AC-susceptibility measurements are done in low magnetic fields, so we expect that the nanoparticles will remain dispersed and in a similar state as probed in the ULF-MRI. When high field measurements are performed, as seen in Fig. 4a, agglomeration and chaining of the nanoparticles may take place altering the magnetic properties.

### Data and materials availability

All data needed to evaluate the conclusions in the paper are present in the paper. Additional data related to this paper may be requested from the authors.
