# Author Correction: Large T_1_ contrast enhancement using superparamagnetic nanoparticles in ultra-low field MRI

**DOI:** 10.1038/s41598-018-31702-0

**Published:** 2018-08-31

**Authors:** Xiaolu Yin, Stephen E. Russek, Gary Zabow, Fan Sun, Jeotikanta Mohapatra, Kathryn E. Keenan, Michael A. Boss, Hao Zeng, J. Ping Liu, Alexandrea Viert, Sy-Hwang Liou, John Moreland

**Affiliations:** 1 National Institute of Standards and Technology, 325 Broadway, CO Boulder, 80305 USA; 2 Nebraska Center for Materials and Nanoscience, University of Nebraska-Lincoln, 855 N.16th St, NE 68588 Lincoln, USA; 3 Department of Physics, University at Buffalo, the State University of New York, 225 Fronczak Hall, NY Buffalo, USA; 4 Department of Physics, University of Texas- Arlington, 502 Yates St, TX 76019 Arlington, USA; 5 Wake Forest Institute for Regenerative Medicine, Wake Forest School of Medicine, NC 27157 Winston-Salem, USA

Correction to: *Scientific Reports* 10.1038/s41598-018-30264-5, published online 08 August 2018

The original version of this Article contained a typographical error in the spelling of the author Jeotikanta Mohapatra, which was incorrectly given as Jeotikatan Mohapatra. This has now been corrected in the PDF and HTML versions of the Article.

